# Combined Dexamethasone Suppression-Corticotropin-Releasing Hormone Stimulation Test in Studies of Depression, Alcoholism, and Suicidal Behavior

**DOI:** 10.1100/tsw.2006.251

**Published:** 2006-10-31

**Authors:** Leo Sher

**Affiliations:** Division of Neuroscience, Department of Psychiatry, Columbia University, New York, NY, USA

**Keywords:** dexamethasone, corticotropin-releasing hormone, DST–CRH test, depression, alcoholism, suicidal behavior

## Abstract

The hypothalamic-pituitary-adrenal (HPA) axis controls the secretion of corticotropin-releasing hormone (CRH), corticotropin (adrenocorticotropic hormone, ACTH), and cortisol. The dexamethasone suppression test (DST) is the most frequently used test to assess HPA system function in psychiatric disorders. Patients who have failed to suppress plasma cortisol secretion, i.e., who escape from the suppressive effect of dexamethasone, have a blunted glucocorticoid receptor response. After CRH became available for clinical studies, the DST was combined with CRH administration. The resulting combined dexamethasone suppression-corticotropin-releasing hormone stimulation (DST–CRH) test proved to be more sensitive in detecting HPA system changes than the DST. There is a growing interest in the use of the DEX-CRH test for psychiatric research. The DEX-CRH test has been used to study different psychiatric conditions. Major depression, alcoholism, and suicidal behavior are public health problems around the world. Considerable evidence suggests that HPA dysregulation is involved in the pathogenesis of depressive disorders, alcoholism, and suicidal behavior. Over the past 2 decades, there has been a shift from viewing excessive HPA activity in depression as an epiphenomenon to its having specific effects on symptom formation and cognition. The study of HPA function in depression, alcoholism, and suicidal behavior may yield new understanding of the pathophysiolgy of these conditions, and suggest new approaches for therapeutic interventions. The combined DEX-CRH test may become a useful neuroendocrinological tool for evaluating psychiatric patients.

## INTRODUCTION

Major depression, alcoholism, and suicidal behavior are public health problems around the world. Major depressive disorder affects approximately 9.9 million Americans or roughly 5% of the population, and is the leading cause of disability in the U.S.[1,2]. The prevalence of depression ranges from 5–9% in primary care and between 40 and 60% of depression treatment occurs in this setting[3,4,5,6,7,8]. From the mid-1980s through the mid-1990s, visits to primary care for depression doubled and the proportion of antidepressant prescriptions written by primary care providers increased from 41–48%[9].

Similarly, alcohol problems, including at-risk drinking, alcohol abuse, and alcohol dependence, are highly prevalent[10,11,12]. The National Institute of Alcohol Abuse and Alcoholism reported a prevalence for alcohol abuse and dependence of 7% in 1992[13]. In one primary care–based study of 19,372 adults, less-severe alcohol problems, such as at-risk drinking, were identified in 19.7% while in population-based studies, hazardous drinking ranged from 18–24% and harmful drinking from 0.3–14%[14,15,16,17,18].

It is important to note that patients with depression frequently have alcohol use disorders. One household survey reported a prevalence of 16% for any alcohol diagnosis in depressed patients[19]. These conditions have been found to coexist in different age, sex, and racial groups[19,20,21,22]. Comorbid alcohol use disorders in depressed patients present diagnostic and management challenges, and may adversely affect the course of depression and its response to standard therapies.

The World Health Organization reported that self-inflicted injuries, including suicide, accounted for more than 800,000 deaths in 2001[23]. If every suicide affects at least six family members or friends, then every year in the world there would be about 5 million new survivors. Suicide rates range from 3.4/100 000 in Mexico to 6.0/100 000 in the U.K., and 34.0/100 000 in the Russian Federation. In the U.S., suicide accounts for about 30,000 deaths per year.

## HPA FUNCTION

The hypothalamic-pituitary-adrenal (HPA) axis controls the secretion of corticotropin-releasing hormone (CRH), corticotropin (adrenocorticotropic hormone), and cortisol[24,25]. CRH is secreted from the paraventricular nucleus of the hypothalamus as well as from extrahypothalamic sites. It acts on the anterior pituitary to cause the release of corticotropin into the bloodstream, where it acts on the adrenal cortex to cause the production and release of cortisol into the bloodstream.

Cortisol has diverse and widespread actions throughout the body and brain[24,25,26,27,28]. It secondarily inhibits corticotropin and CRH release via negative feedback, although it may augment CRH release in the amygdala. Feedback inhibition is mediated via low-affinity glucocorticoid receptors (GRs) and high-affinity mineralocorticoid receptors (MRs). In the brain, MRs are located primarily in the hippocampus. GRs are more widely distributed in the hypothalamus, pituitary, cortex, and elsewhere. Species from humans to the most ancient organisms share components of the HPA axis.

Release of CRH from the hypothalamus is influenced by stress, by blood levels of cortisol, and by the sleep/wake cycle[26,27,28]. In healthy persons, levels of cortisol rise rapidly after waking, reaching a peak within 30–45 min. They then gradually diminish over the day, rising again in late afternoon. Cortisol levels fall in late evening, reaching a trough during the middle of the night. The circadian pattern of cortisol release is controlled by the suprachiasmatic nucleus (SCN) of the hypothalamus, also known as the “body clock”. Nerve signals from the SCN cause the paraventricular nucleus of the hypothalamus to release pulses of CRH roughly once per hour, resulting in HPA axis activation and cortisol release. There are also direct links between the SCN and the adrenal gland itself (bypassing the HPA axis) through sympathetic nerve fibers, causing the adrenal gland to become more sensitive to corticotropin stimulation during the morning and further adding to the circadian pattern of cortisol release throughout the day.

## THE HPA AXIS AND DEPRESSION

Over the past 2 decades, there has been a shift from viewing excessive HPA activity in depression as an epiphenomenon to its having specific effects on symptom formation and cognition[29,30,31]. Studies have demonstrated that hypercortisolism is involved in the pathogenesis of depressive disorders. Researchers have suggested that HPA dysregulation is involved in causality of depression and have proposed that antidepressants may act through normalization of pathologic HPA axis changes[30,31,32].

The following clinical observations have been made in patients with depression: (a) the number of cortisol and corticotropin secretory pulses is increased, which is also reflected in elevated urinary cortisol production rates; (b) levels of CRH in the cerebrospinal fluid are elevated; (c) the number of CRH-secreting neurons in limbic brain regions is increased; (d) the number of CRH binding sites in the frontal cortex is reduced secondary to increased CRH concentration; (e) the dexamethasone suppression test (DST), CRH stimulation test, combined DST-CRH stimulation test, and other neuroendocrine function tests indicate HPA dysregulation[29,30,31,32,33,34].

Increased cortisol secretion caused by major and minor stressful events may contribute to the development of depressive disorders in vulnerable individuals[35]. Therefore, one of the goals of prevention of such stress-related disorders is to help individuals to be more competent in managing their behavior and emotions in reaction to the negative aspects of their environment.

## THE DEX-CRH TEST

The DST is the most frequently used test to assess HPA system function in psychiatric disorders[36,37,38]. This neuroendocrine test consists of the administration of a low dose of dexamethasone at 11 p.m. and the measurement of cortisol levels at one or more time points on the following day. Dexamethasone acts at corticotrophic cells by binding to glucocorticoid receptors that, through negative response elements in the promoter region of the proopiomelanocortin (POMC) gene, inhibit the expression of this gene and, subsequently, the synthesis and secretion of ACTH. In turn, the suppression of ACTH through dexamethasone prompts a decrease of corticosteroid secretion (cortisol in humans and corticosterone in rats and mice). In healthy controls, the appropriate response is a suppression of ACTH and cortisol. The parameter that is measured by this test is the capacity of the glucocorticoid receptors of pituitary corticotrophs to exert a negative regulatory effect on the release of ACTH and, consequently, cortisol. Thus, patients who have inappropriately failed to suppress plasma cortisol secretion, i.e., who escape from the suppressive effect of dexamethasone, have a blunted glucocorticoid receptor response. This impairment can be primary, i.e., genetically derived, or secondary, i.e., caused by an enhanced ACTH and cortisol secretion, which leads to glucocorticoid receptor desensitization[36,37,38].

After CRH became available for clinical studies, the DST was combined with CRH administration[30,39,40,41,42]. In this test, patients are pretreated with a single dose of dexamethasone at 11 p.m. and receive CRH intravenously at 3 p.m. the following day. The resulting DST–CRH test proved to be much more sensitive in detecting HPA system alterations than the DST[30,39,40,41,42]. Several studies show that depressed patients pretreated with dexamethasone react by an exaggerated ACTH and cortisol response when a fixed dose of 100 µg human CRH is injected. In the DEX-CRH test, dexamethasone, which does not bind to corticosteroid-binding globulins, exerts HPA suppression primarily at the level of the pituitary corticotrophs, but does not suppress hypothalamic CRH nor vasopressin as effectively as endogenous corticosteroids. The subsequent decrease in cortisol and the failure of dexamethasone to compensate for decreased cortisol levels in the nervous tissue simulates the effects of a partial or transient adrenalectomy. In response, central neuropeptides that activate ACTH secretion, mainly CRH and vasopressin, are secreted. Since hypothalamic vasopressin is higher in depressed patients compared with controls, in depressed dexamethasone-pretreated patients, secretion of ACTH in response to exogenous CRH would be greater than in controls because of the synergistic interaction of the administered CRH bolus with a large amount of vasopressin present at the corticotrophs.

## THE DEX-CRH TEST AND DEPRESSION

There is a growing interest in the use of the DEX-CRH test for psychiatric research around the world[43,44,45]. The DEX-CRH test has been used to study depression, suicidal behavior, alcoholism, and other psychiatric conditions.

Depressed patients release significantly more cortisol and ACTH after dexamethasone and additional CRH in comparison with age-matched controls[43]. This hormonal release pattern supports the assumption that depressed patients are prone to an altered glucocorticoid feedback regulation during the acute illness episode. In some DEX-CRH studies, patients are maintained on antidepressants and, in some studies, patients are medication free. How much do antidepressants influence HPA regulation and the DEX-CRH test results? The DEX-CRH test appears to be the most sensitive tool to detect depression-related changes in the HPA axis[43]. Changes in HPA axis reactivity in this test were correlated with changes in depressive symptomatology. Normalization of the DEX-CRH test was shown to anticipate or parallel response to antidepressant treatment[43,46,47,48]. It has been suggested that antidepressant treatment does not appear to affect test outcome in the absence of clinical response[46]. Kunzel et al.[46] did not find an association of presence or absence of antidepressant treatment, the type of antidepressant treatment, or of the number of previous ineffective antidepressant treatment attempts before hospitalization and the results of the DEX-CRH test. However, it has been shown that mirtazapine effectively reduces the overshoot of cortisol and ACTH during the DEX-CRH test, both in treatment responders and nonresponders within 1 week[49]. The authors suggest that mirtazapine rapidly attenuates HPA axis hyperactivity in depressed patients via direct pharmacoendocrinological effects and this amelioration of HPA system dysregulation is not necessarily related to clinical improvement. In another study, both 6- and 12-week fluvoxamine treatments were associated with a significant and robust reduction of ACTH and cortisol response to the DEX-CRH test in patients with borderline personality disorder[50]. These results were independent of psychiatric comorbidity including comorbidity with major depression. It has also been reported that imipramine, clomipramine, and desipramine have substantial effects on the HPA axis[51].

The existing body of evidence suggests that antidepressants may affect HPA function and the DEX-CRH test results. Antidepressants may have effects that are independent of their effects on biogenic amine metabolism or receptors and which produce normalization of initial HPA dysregulation. However, it is not clear how much antidepressants affect the HPA axis and the results of the DEX-CRH test in treatment nonresponders. It is interesting to hypothesize that different antidepressants produce different effects on the HPA function and the DEX-CRH test results.

## THE DEX-CRH TEST AND ALCOHOLISM

Alcoholism is associated with abnormalities in HPA function. Animal studies shows that chronic alcohol consumption induces a persistent impairment in the ability of the HPA axis to respond to stress[52,53]. Human studies have demonstrated that chronic alcohol consumption decreases brain and pituitary beta-endorphin activity as well as pituitary ACTH release[54,55]. Subjects with alcohol dependence exhibit lower plasma ACTH and higher plasma cortisol levels under basal conditions and blunted pituitary ACTH and cortisol responses to exogenous CRH and physiological and psychological stressors. Nonalcoholic subjects with a family history of alcoholism exhibit lower plasma ACTH and beta-endorphin as well as lower ACTH, cortisol, and beta-endorphin responses to psychological stress and CRH stimulation[56,57]. This suggests that in children of alcoholics, alterations in the mechanisms that regulate HPA axis activity predate the development of alcohol dependence and may be considered inherited traits. Therefore, studies of the HPA system in individuals at risk for alcoholism may help us to understand the neurobiological mechanisms of predisposition to alcoholism. The combined DST-CRH test may be a useful tool for these studies.

## THE DEX-CRH TEST AND SUICIDAL BEHAVIOR

An association between suicidal behavior and hyperactivity of the HPA axis has been suggested[58,59,60,61,62,63]. The two most consistent findings regarding the HPA axis relate to violence of attempts and eventual suicide completion. Eventual suicides are reported to have higher cortisol levels after being administered dexamethasone than nonsuicides, and HPA axis hyperactivity at baseline may increase the odds of an eventual suicide as much as 14-fold[59,62]. Patients with a history of violent suicide attempt(s) are reported to have higher levels of urinary-free cortisol, higher serum cortisol levels in response to 5HTP, and higher rates of DST nonsuppression compared with patients without such history[58,59,61,64,65,66,67]. It has recently been shown that suicidal behavior in depressed patients, including past and recent suicide attempts as well as suicidal ideation, was associated with a lower ACTH and cortisol response in the combined DEX-CRH test, with lowest hormone levels observed in patients with a recent suicide attempt[68].

## CONCLUSIONS

The study of HPA function in depression, alcoholism, and suicidal behavior may yield new understanding of the pathophysiolgy of these conditions and suggest new approaches for therapeutic interventions. The combined DEX/CRH test may become a useful neuroendocrinological tool for evaluating psychiatric patients.
